# *Annona coriacea* Mart. Fractions Promote Cell Cycle Arrest and Inhibit Autophagic Flux in Human Cervical Cancer Cell Lines

**DOI:** 10.3390/molecules24213963

**Published:** 2019-11-01

**Authors:** Izabela N. Faria Gomes, Renato J. Silva-Oliveira, Viviane A. Oliveira Silva, Marcela N. Rosa, Patrik S. Vital, Maria Cristina S. Barbosa, Fábio Vieira dos Santos, João Gabriel M. Junqueira, Vanessa G. P. Severino, Bruno G Oliveira, Wanderson Romão, Rui Manuel Reis, Rosy Iara Maciel de Azambuja Ribeiro

**Affiliations:** 1 Experimental Pathology Laboratory, Federal University of São João del Rei—CCO/UFSJ, Divinópolis 35501-296, Brazil; izabela.faria.tk@hotmail.com (I.N.F.G.); patrikdasilvavital@gmail.com (P.S.V.); 2 Molecular Oncology Research Center, Barretos Cancer Hospital, Barretos 14784-400, Brazil; renatokjso@gmail.com (R.J.S.-O.); vivianeaos@gmail.com (V.A.O.S.); nr.marcela@gmail.com (M.N.R.); 3 Laboratory of Cell Biology and Mutagenesis, Federal University of São João del Rei—CCO/UFSJ, Divinópolis 35501-296, Brazil; mariacristina@ufsj.edu.br (M.C.S.B.); fabiosantos@ufsj.edu.br (F.V.d.S.); 4 Special Academic Unit of Physics and Chemistry, Federal University of Goiás, Catalão 75704-020, Brazil; jgmjunqueira@gmail.com (J.G.M.J.); vanessa.pasqualotto@gmail.com (V.G.P.S.); 5 Petroleomic and forensic chemistry laboratory, Department of Chemistry, Federal Institute of Espirito Santo, Vitória, ES 29075-910, Brazil; brunoliveir_ra20@msn.com (B.G.O.); wandersonromao@gmail.com (W.R.); 6 Life and Health Sciences Research Institute (ICVS), Medical School, University of Minho, 4710-057 Braga, Portugal; 7 3ICVS/3B’s-PT Government Associate Laboratory, 4710-057 Braga, Portugal

**Keywords:** natural compounds, cervical cancer, autophagy, cell cycle arrest

## Abstract

Plant-based compounds are an option to explore and perhaps overcome the limitations of current antitumor treatments. *Annona coriacea* Mart. is a plant with a broad spectrum of biological activities, but its antitumor activity is still unclear. The purpose of our study was to determine the effects of *A. coriacea* fractions on a panel of cervical cancer cell lines and a normal keratinocyte cell line. The antitumor effect was investigated in vitro by viability assays, cell cycle, apoptosis, migration, and invasion assays. Intracellular signaling was assessed by Western blot, and major compounds were identified by mass spectrometry. All fractions exhibited a cytotoxic effect on cisplatin-resistant cell lines, SiHa and HeLa. C3 and C5 were significantly more cytotoxic and selective than cisplatin in SiHa and Hela cells. However, in CaSki, a cisplatin-sensitive cell line, the compounds did not demonstrate higher cytotoxicity when compared with cisplatin. Alkaloids and acetogenins were the main compounds identified in the fractions. These fractions also markedly decreased cell proliferation with p21 increase and cell cycle arrest in G2/M. These effects were accompanied by an increase of H2AX phosphorylation levels and DNA damage index. In addition, fractions C3 and C5 promoted p62 accumulation and decrease of LC3II, as well as acid vesicle levels, indicating the inhibition of autophagic flow. These findings suggest that *A. coriacea* fractions may become effective antineoplastic drugs and highlight the autophagy inhibition properties of these fractions in sensitizing cervical cancer cells to treatment.

## 1. Introduction

Cervical cancer is the fourth most common cancer among women worldwide, accounting for 7.5% of all female cancer deaths [1]. The number of diagnosed cases is about twice as high in developing countries, such as Brazil, where cervical cancer corresponds to the third most common type among Brazilian women [2]. Despite the high incidence, cervical cancer is one of the tumor types that present significant potential for prevention [3]. Nevertheless, many cases are still diagnosed at an advanced stage, and the therapeutic options are limited [4]. So far, platinum-based chemotherapy remains the only anticancer approach that has improved the results in recurrence and metastatic cervical cancer [5]. However, cisplatin demonstrated extensive side effects, such as myelosuppression and nephrotoxicity, as well as problems related to the resistance and relapse of the disease [5]. Currently, results with molecular-targeted therapies constitute potential alternatives, but the clinical outcomes are still in progress [6]. In this context, natural compounds offer an exciting option to explore and maybe overcome the treatment limitations for cervical cancer.

Autophagy is a homeostatic biological process that maintains cell survival by recycling organelles and molecules, but its role in cancer is still unclear [7]. Autophagic process is activated in many tumors and, when inhibited, can lead to cell death or survival, depending on the tissue type, tumor grade, and therapy [8]. In cervical cancer, autophagy activation is reported as a target of paclitaxel (Taxol), a relevant natural product in cancer chemotherapy resistance [9]. Autophagy targeting has been recognized as a novel therapeutic approach. So far, the establishment of news autophagy modulators is required for cancer treatment [10].

Annonaceae is a common Brazilian plant family, with 29 genera and approximately 386 species [11]. Some species of Annonaceae, a family of plants widely distributed in Brazil, have been related by their biological activity as an anticancer, analgesic, and antimicrobial [12,13]. *Annona coriacea* Mart., a member of the Annonaceae family, is one of the endemic species of the Brazilian Cerrado. It is popularly known as “araticum-liso”, “marola”, or “araticum do campo” [14]. Among the biological activities already reported for the species are analgesic, anti-inflammatory, carminative, and anthelmintic activity [15]. Recently, methanolic extract of *A. coriacea* seeds exhibited cytotoxicity activity against some cancer cell lines [16]. Although the advantage of obtaining and developing a therapy from leaves rather than other plant parts is clear, potential cytotoxicity activity from *A.coriacea* leaves remains unknown.

The goal of the current study was to evaluate the antineoplastic activity of seven fractions of leaves of *A. coriacea* in human cervical cancer cell lines. We analyzed several biological effects, such as cytotoxicity, proliferation, cell death by apoptosis and autophagy, cell migration, and tumorigenesis, to explore their potential in cervical cancer treatment.

## 2. Results

### 2.1. Anonna coriacea Mart. Fractions Contain Acetogenins and Alkaloids in Their Constitution

Analysis of the Electrospray Ionization Fourier Transform Ion Cyclotron Resonance Mass Spectrometry (ESI (-) FT-ICR MS) profile of *A. coriacea* fractions suggests the presence of acetogenins as bulatacin, annonacin, annohexocin, anomuricin E, and coriaheptocinin magnification of 500 to 700 m/z regions in both fractions (C3 and C5). The m/z values of the main molecules found in C3 and C5 are shown in Table 1. Supplementary Table S1 summarizes the major features of the seven fractions isolated.

### 2.2. A. coriacea Fractions Promote Cytotoxicity in a Dose- and Time-Dependent Manner in Cervical Cancer Cells Lines

In order to determine the cytotoxicity effects of *A. coriacea* fractions on human cervical cancer cell lines, the cells were cultured and treated with various concentrations of *Annona coriacea* fractions or cisplatin (CIS), respectively, for 72 h, followed by the use of an MTS assay to analyze the cell viability. As shown in Table 2, of the seven fractions used, five reached the IC_50_ ( half maximal inhibitory concentration) for the three tested cell lines, and fractions C2 and C4 did not affect cell viability. The IC_50_ values decreased as the concentration of fraction increased, suggesting a dose-dependent manner. The IC_50_ values for the CaSki cell line ranged from 3.6 to 21.4 µg/mL, from 4.1 to 12.9 µg/mL in HeLa, and from 5.1 to 16.1 µg/mL in the SiHa cell line (Table 2). Notably, for the HeLa and SiHa cell lines, the cisplatin-resistant cell lines, all fractions showed a lower IC_50_ than cisplatin (Table 2). However, for CaSki cells, a cisplatin-sensitive cell line, the compounds did not demonstrate higher cytotoxicity as compared with cisplatin.

Thus, based on these results, we continued the studies with the two most cytotoxic and selective fractions, C3 and C5 (Table 3). For both fractions, the IC_50_ in HaCaT cells was greater than those observed for cisplatin. Regarding the selectivity indexes, C5 was more selective than C3 in all cell lines tested. Moreover, the fractions in the cisplatin-resistant cell lines (HeLa and SiHa) showed better selectivity than cisplatin. However, for the cisplatin-sensitive cell line, the compounds did not show better selectivity indexes when compared with cisplatin. The same effect was observed over time (from the 3 until 72 h) for C3 and C5 (see Table S2 in the Supplementary Materials).

**Figure 1:**
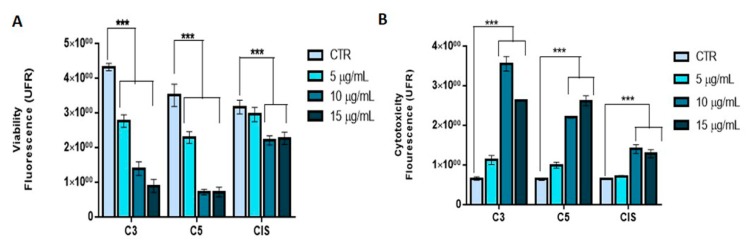
Cytotoxicity in SiHa cells. (**A**) Cell viability measured after 24 h of exposure in SiHa cells. (**B**) Cell cytotoxicity measured after 24 h of exposure in SiHa cells. There was an increase in cytotoxicity and a decrease in viability in a dose-dependent manner (*p* < 0.0001). C3: Ethyl acetate fraction; C5: Fraction enriched in acetogenin; Cis: cisplatin. *** Indicates a statistical difference between groups. UFR: Relative unit of fluorescence.

We selected SiHa cells to evaluate the mechanism of action of the C3 and C5 fractions since they are resistant to cisplatin and because these treatments showed greater cytotoxicity and selectivity to this cell line (Figure 1A,B).

### 2.3. A. coriacea Fractions Inhibited Cell Proliferation and Invasion, and Induced Cell Cycle Arrest in Cervical Cancer Cell Lines

**Figure 2:**
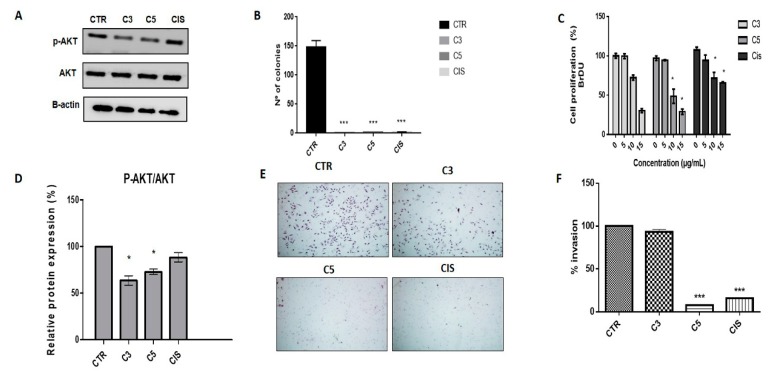
Cell proliferation and invasion upon C3 and C5 treatment (5µg/mL) in SiHa cells (**A**) Western blotting of phospho-AKT (protein kinase B) upon C3 and C5 treatment. (**B**) Number of colonies in the soft agar assay performed for 45 days. (**C**) BrdU incorporation after C3 and C5 treatment (*p* < 0.0001) in SiHa cells. (**D**) Densitometry of p-AKT. (**E**) Invasion inhibition through C3 and C5 treatments in SiHa cells. (**F**) Percentage of invasion cells in SiHa cells (*** *p* < 0.0001; * *p* < 0.05). * Indicates statistical difference between the treatments). C3: Ethyl acetate fraction; C5: Fraction enriched in acetogenin; Cis: cisplatin.

We analyzed the effect of C3 and C5 fractions on cell proliferation. The C3 and C5 fractions reduced AKT phosphorylation (Figure 2A,D) and also promoted a reduction in more than 90% of the number of colonies in anchorage-independent growth in comparison to the control (Figure 2B). Moreover, C5 was able to reduce BrdU incorporation significantly (Figure 2C). Using the matrigel invasion assay, we observed that the C5 fraction significantly inhibited cell invasion in SiHa cells. Also, C5 demonstrated higher invasion inhibition when compared with cisplatin (Figure 2E,F).

**Figure 3:**
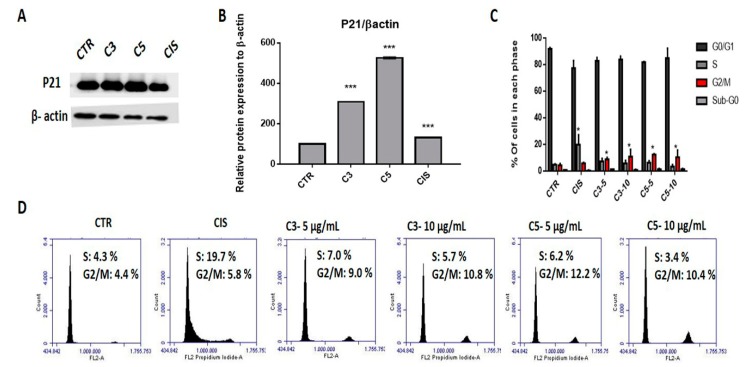
Cell cycle alterations in SiHa cells after exposure to C3 and C5 compounds (**A**) Western blot of p21 in SiHa cells upon C3, C5, and cisplatin treatments. (**B**) Densitometry of p21. (**C**) Cell cycle profile in SiHa cells. (**D**) Cell cycle phase distribution after treatment with C3 and C5. (*** *p* < 0.0001; * *p* < 0.05). C3: Ethyl acetate fraction; C5: Fraction enriched in acetogenin; Cis: cisplatin; DMSO: dimethylsulfoxide.

We further analyzed the expression of proteins involved in the cell cycle. The C3 and C5 treatments increased p21 expression (Figure 3A,B). Regarding the cell cycle, we observed that both C3 and C5 promoted cell cycle arrest in the G2/M phase in SiHa cells after treatment with 5 and 10 µg/mL of each fraction (Figure 3C,D).

### 2.4. Annona coriacea Fractions Promote Cytotoxic Effects by DNA Damage but Do Not Induce Apoptosis

**Figure 4:**
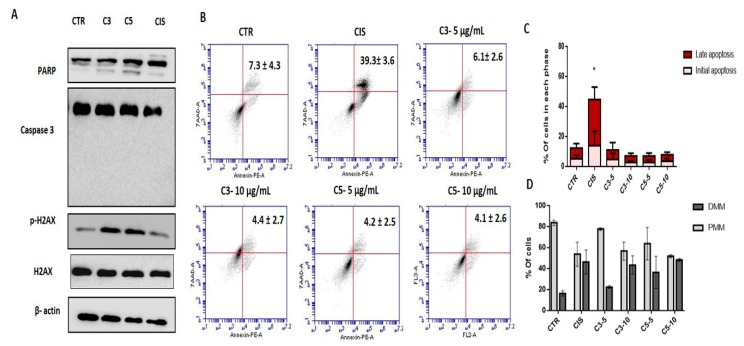
Apoptosis evaluation in SiHa cells upon C3 and C5 compounds. (**A**) Western blot of PARP (Poly (ADP-ribose) polymerase), caspase 3, and H2AX (H2A histone family member X) proteins (**B**) Flow cytometry for SiHa cells. (**C**) Comparison of apoptotic cells upon C3 and C5 treatment. There was a significant increase for cells in apoptosis only for cisplatin (CIS) * *p* = 0.0282 (**D**) Depolarization of the mitochondrial membrane after treatment with C3 and C5 and cisplatin in the SiHa cell line. C3: Ethyl acetate fraction; C5: Fraction enriched in acetogenin; Cis: cisplatin; DMM: Depolarized mitochondrial membrane; PMM: Polarized mitochondrial membrane. *p* < 0.05). C3: Ethyl acetate fraction; C5: Fraction enriched in acetogenin; Cis: cisplatin.

We also analyzed the protein expression of poly (ADP-ribose) polymerase (PARP) and caspase 3 by Western blot. We observed that C3 and C5 treatment increased cleavage of PARP but not caspase 3 (Figure 4A) As opposed to the action of cisplatin, exposure to C3 and C5 fractions did not induce apoptosis (Figure 4B,C). Additionally, the mitochondrial membrane potential was analyzed, and we verified that at higher concentrations, C3 and C5 induce mitochondrial membrane depolarization similar to cisplatin (Figure 4D). Taken together, these results suggest that apoptosis is not the main mechanism of cell death induced by *A. coriacea* Mart. fractions.

**Figure 5:**
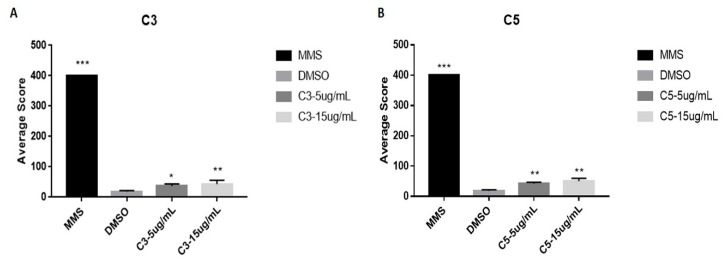
DNA damage evaluation in HeLa cells upon C3 and C5 compounds. (**A**) Genotoxic damage induced by C3. (**B**) Genotoxic damage induced by C5. Data are representative of three experiments. Error bars represent SD. C3: Ethyl acetate fraction; C5: Fraction enriched in acetogenin; Cis: cisplatin; C3: Ethyl acetate fraction; C5: Fraction enriched in acetogenin; Cis: cisplatin; MMS: Methyl methane sulphonate, DMSO: dimethylsulfoxide. *** *p* < 0.0001; ** *p* < 0.01, * *p* < 0.05).

To better understand the cytotoxic effects of *A. coricea* fractions, we performed the comet assay on HeLa cells, which has a response profile to both fractions that is similar to SiHa. The results showed that *A. coriacea* fractions significantly increased on average the damage score in comparison with the control (Figure 5A,B). Moreover, in agreement with these results, both fractions increased the expression of H2AX phosphorylation, suggesting their role in DNA damage (Figure 4A).

### 2.5. A. coriacea Fractions Promote Autophagy Flux Inhibition in Cervical Cancer Cell Lines

**Figure 6:**
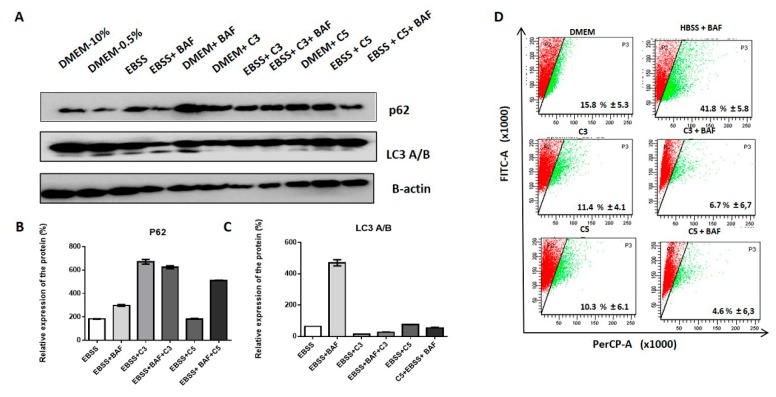
Analysis of the involvement of *A. coriacea* fractions in autophagy. (**A**) Analysis of the expression of proteins involved in the autophagic flux in SiHa cells. (**B**) Densitometry of p62. (**C**) Densitometry of LC3 B/A (Microtubule-associated protein 1A/1B-light chain 3). (**D**) Acridine orange staining in SiHa cells. There was a reduction in the percentage of formation of acid vesicles, evidenced by a reduction of the fluorescent green signal after treatment with the fractions (*p* < 0.05). HBSS: Hank’s balanced salt solution; EBSS: Earle’s balanced salt solution; C3: Ethyl acetate fraction; C5: Fraction enriched in acetogenin; Cis: cisplatin. BAF: Bafilomycin.

To explore the role of C3 and C5 in autophagic flux, we analyzed by Western blot the expression of critical proteins for the autophagic pathway. Interestingly, we found these fractions induced an increase in p62 (Figure 6A–C). Furthermore, by acridine orange staining, we verified that C3 and C5 fractions produced a reduction of acidic vesicles (Figure 6D), suggesting that less autophagosome formation could be associated with inhibited autophagy initiation. These findings suggest that *A. coriacea* fractions may inhibit the initiation steps of autophagy.

## 3. Discussion

Cisplatin is the most frequent chemotherapy agent used for metastatic and refractory cervical tumors; yet, it has demonstrated high recurrence rates due to its high toxicity and resistance [5]. In this context, it is necessary to develop new and less toxic therapeutic approaches. In the present study, we observed that *Annona coriacea* fractions promoted a cytotoxic effect, cell cycle arrest, and inhibit autophagy as well as invasion.

*Annona coriacea* compounds were able to promote cytotoxicity in cervical cancer cell lines in a dose- and time-dependent-manner. It can also be inferred that these compounds are considered pharmacologically active according to the recommendation of the Institute National Cancer Institute (NCI) for IC_50_ values less than 30 µg/mL [17]. The results are in accordance with previous studies that reported the antiproliferative activity of *Annonaceae* [12,18]. Our results showed that C3 and C5 are selective for tumor cells when compared with normal skin keratinocyte. One limitation of our study is the lack of an ideal normal cervix cell line counterpart to evaluate the selectivity index. Previous studies have considered that a value greater than or equal to 2.0 is an interesting selectivity index, which means that the compound is more than twice more cytotoxic to the tumor cell line as compared with the normal cell line [19].

Among the compounds tested, C3 and C5 were those that showed higher cytotoxicity concerning the other compounds. Moreover, C3 and C5 showed more selectivity when compared with cisplatin in the cisplatin-resistant cell lines. These fractions are rich in acetogenins and alkaloids, as previously described in the *Annonaceae* family [11,14,20,21,22,23,24,25,26,27,28,29,30,31,32,33,34,35,36,37,38,39,40,41,42]. Chemical studies on *Annonaceae* species have identified a large number of acetogenins and alkaloids that possess great biological and pharmacological potential due to their antitumor, cytotoxic, and apoptosis-inducing activities [43]. Many of these metabolites have already been described as acting on crucial enzymes for cell division, such as topoisomerases and the deregulation of the phosphorylative chain by inhibition of the mitochondrial I complex [44]. Taken together, these results suggest that the phytochemical constituents of C3 and C5, among them acetogenins and alkaloids, might contribute substantially to the antineoplastic effect of *A. coriacea* fractions in cervical cancer.

Proliferation inhibition is another characteristic attributed to natural compounds [45,46]. In accordance, we also observed that C3 and C5 treatment promoted a significant increase in the p21 levels, a key regulator of the cell cycle, correlating with G2/M arrest as well as a decrease in BrdU incorporation and p-AKT. Cell cycle arrest in the G2/M transition can be attributed to cytoskeletal disorganization by inhibition of the mitotic spindle [47]. Also, as previously reported, cisplatin treatment induced p53 accumulation and upregulated P21 expression as well as cell cycle arrest in the G1/S phase [48]. Many alkaloids are reported as both mitotic spindle inhibition promoters, such as vinblastine, and as promotors of the inhibition of topoisomerases, such as liriodenine [49,50]. Moreover, cell cycle disruption and repair enzyme overexpression can be attributed to DNA damage [51].

The invasion and clonogenic activity of C3 and C5 were also evaluated in cervical cancer cell lines. The results demonstrated that C3 and C5 treatment significantly reduced the number and size of colonies, as well as the number of invasive cells. A nanoformulation based on curcumin, a natural acetogenin as founded in C3 and C5, has also shown promising results in reducing invasion rates and colonies formed for the SiHa cell line [52]. In this way, it can be inferred that *A. coriacea* fractions could play an inhibitory role in invasion and metastasis processes.

Apoptosis has been described as a key mechanism for the antitumor activity of natural products [53]. Our findings suggest that *A. coriacea* fractions do not induce apoptosis, although we found alterations of the PARP cleavage and H2AX activity involved in DNA repair. The comet assay showed that C3 and C5 promoted an increase in DNA damage. Thus, these data together provide evidence that the cytotoxicity of *A. coricea* fractions can be related to DNA damage directly.

Regarding the autophagic flux, we found decreased expression of LC3 cleavage, increased p62 levels, and negative labeling of acidic vesicles by acridine orange. In accordance with our results, AKT downregulation has already reported as being involved in autophagy and apoptosis through the beclin-1 block, and the PI3K/AKT/mTOR pathway has been implicated as one of the principals of autophagy pathways in gynecological cancers [54]. Moreover, some studies have shown that G2/M cell cycle arrest is a target of autophagy inhibition [55]. Thus, these results indicate that C3 and C5 could inhibit the initiation of autophagy or even the vesicular traffic in cervical cancer.

## 4. Materials and Methods

### 4.1. Plant Material

The leaves of *A. coriacea* Mart. were collected in May 2010, at the Federal University of Goias, Catalão, GO, Brazil (18°09′16.4″ S; 47°55′43.2″ W). Dr. Helder N. Consolaro from the Academic Unit of Biotechnology, Federal University of Goias, Catalão, GO, Brazil carried out the identification, and a voucher specimen (no. 47919) was deposited at the Herbarium of Integrated Laboratory of Zoology, Ecology, and Botany in the same university. Registration was made in National System for Management of Genetic Heritage and Associated Traditional Knowledge (SISGEN): A11AE20.

### 4.2. Preparation of Extracts

Leaves (619 g) from *A. coriacea* were subjected to exhaustive maceration in EtOH (Sigma Aldrich, # 459836) at room temperature. The filtered material was concentrated in a rotary evaporator under reduced pressure at 40 °C to yield the ethanolic extract of the leaves (57.5 g; C1). The ethanolic extract of the leaves was solubilized in MeOH/H2O (3:7, *v*/*v*) and subjected to liquid-liquid extraction with n-hexane (Sigma Aldrich # 650552) and ethyl acetate (Sigma-Aldrich, San Luis, EUA #270989; EtOAc). After the evaporation of the solvent under reduced pressure, fractions were obtained: Hexane (12.3 g; C2), EtOAc (20.5 g; C3), and hydroalcoholic (5.0 g; C4). From the separation of C3, fraction C3.3.4.2 (3.3 g; C5) resulted. The ethanolic extract of leaves was subjected to an acid-base extraction resulting in neutral (5.2 g; C6) and alkaloidal (0.32 g; C7) fractions.

### 4.3. Electrospray Ionization Fourier Transform Ion Cyclotron Resonance Mass Spectrometry (ESI (−) FT-ICR MS)

To identify the main chemical compounds present in *A. coriacea* Mart. fractions, negative electrospray ionization coupled to Fourier transform ion cyclotron resonance mass spectrometry (ESI (-)-FT-ICR MS) analysis was performed as described in the literature [56]. Briefly, 10 µL of each fraction was dissolved in 1000 µL of methanol/toluene (Sigma-Aldrich, # 1.06018; #650579; 50% *v*/*v*). Afterward, the solution was basified with 4 µL of NH_4_OH (Vetec Fine Chemicals Ltda, Brazil, # 60REAQMO002448). Samples were directly infused at a flow rate of 4.0 mL/min into the ESI (−) source.

The mass spectrometer (model 9.4 T Solarix, Bruker Daltonics, Bremen, Germany) was set to negative ion mode, ESI (−), over a mass range of *m*/*z* 150–1500. The ESI source conditions were as follows: A nebulizer gas pressure of 1.5 bar, a capillary voltage of 3.8 kV, and a transfer capillary temperature of 200 °C. The ions’ time accumulation was 2 s. ESI (−) FT-ICR mass spectra were acquired by accumulating 32 scans of time-domain transient signals in four mega-point time-domain data sets. All mass spectra were externally calibrated using NaTFA (m/z from 200 to 1200). A resolving power, m/∆m50% = 500,000 (in which m/∆m50% is the full-peak width at the half-maximum peak height of m/z 400), and mass accuracy of <1 ppm provided the unambiguous molecular formula assignments for singly charged molecular ions.

The mass spectra were acquired and processed using data analysis software (Bruker Daltonics, Bremen, Germany). Elemental compositions of the fractions were determined by measuring the m/z values. The proposed structures for each formula were assigned using the Chemspider (www.chemspider.com) database. The degree of unsaturation for each molecule can be deduced directly from its DBE (double bond equivalent) value according to the equation, DBE = c − h/2 + n/2 + 1, where c, h, and n are the numbers of carbon, hydrogen, and nitrogen atoms, respectively, in the molecular formula.

### 4.4. Cell Lines and Cell Culture

CaSki, HeLa, SiHa cells (ATCC catalog number CRL-1550, CCL-2, and HTB-35, respectively), and one normal keratinocytes cell line (HaCaT) were kindly provided by Dr. Luisa Villa. All the cell lines were maintained in Dulbecco’s modified eagle’s medium (DMEM1X, high glucose; Gibco, Invitrogen, Grand Island, NY, USA) supplemented with 10% fetal bovine serum (FBS, Gibco, Invitrogen, #26140079) and 1% penicillin/streptomycin solution (P/S, Gibco, #15140122), at 37 °C and 5% CO_2_2. Authentication of cell lines was performed by the Department of Molecular Diagnostics, Barretos Cancer Hospital. Genotyping confirmed the identity of all cell lines, as previously reported [57]. Moreover, all cell lines were tested for mycoplasma through the MycoAlert™ Mycoplasma Detection Kit (Lonza), following the manufacturer’s instructions.

### 4.5. Drugs

Cisplatin was obtained from Sigma Aldrich (#479306), and its stock solution was prepared in NaCl 0.9%. The stocks solutions of all the fractions were prepared in dimethyl sulfoxide (Sigma-Aldrich, #472301, DMSO). All solutions were stored at −20 °C. Cisplatin was subsequently prepared as intermediate dilutions in DMSO to obtain an equal quantity of DMSO (1% final concentration) in each of the conditions studied. In all experimental conditions, the drugs were diluted in 0.5% FBS culture medium (DMEM-0.5% FBS). Vehicle control (1% DMSO, final concentration) was also used in all experiments.

### 4.6. Cell Viability and Selectivity Assay

The cell viability and selectivity were performed by MTS (-(4,5-dimethylthiazol-2-yl)-5-(3-carboxymethoxyphenyl)-2-(4-sulfophenyl)-2H-tetrazolium, Promega, Madison, WI, # G3581) as previously described [57]. To determine the IC_50_ values, the cells were seeded into 96-well plates at a density of 5 × 10^3^ cells per well and allowed to adhere overnight in DMEM-10% FBS. Subsequently, the cells were treated with increasing concentrations of the *A. coriacea* fractions (0, 1, 2.5, 5, 7.5, 10, 20, and 25 µg/mL) and cisplatin (0, 1, 3,6, 9, 12, 15, and 18 µg/mL) diluted in DMEM-0.5% FBS for 72 h and analyzed over time (3, 6, 12, 16, 24, and 36 h) [12]. The selectivity index (SI) of *A. coriacea* compounds were determined as previously reported [19]. The SI of the more cytotoxic fractions (C3 and C5) was calculated by the ratio of the IC_50_ values of the treatments in a normal cell line (HaCaT) to those in the cancer cell lines.

The cytotoxicity and viability were also assessed by ApoTox-Glo (Promega, Madison, WI, # G6320). The results are expressed as the mean viable cells relative to DMSO alone (considered as 100% viability) ± SD. For the kinetics assay, the results were calibrated to the starting viability (time 0 h, considered as 100% of viability) and are expressed as the means ± SD. The IC_50_ concentration was calculated by nonlinear regression analysis using GraphPad Prism software. Both assays were done in triplicate at least three times.

### 4.7. Proliferation Assay

The ELISA-BrdU assay was performed as previously described [58]. Cells were seeded at 5 × 10^3^ densities per well and treated with increasing doses (5, 10, and 15 µg/mL) of the fractions and cisplatin. After 24 h, cell proliferation was detected by the ELISA-BrdU Kit (Roche, Basel, Switzerland #11647229001), following the manufacturer’s specifications.

### 4.8. Cell Cycle Analysis

Cell cycle distribution was analyzed by flow cytometry using propidium iodide (PI) DNA staining [59]. The cells were plated in a six-well plate at a density of 2 × 10^5^ cells per well, and the next day, the cells were treated with fixed concentrations of the fractions and cisplatin. After 24 h, the cells were disrupted and incubated with 40 µg mL^−1^ of PI (Cycle Test Plus BD solution, # 340242) for 10 min at 37 °C, 5% CO_2_, as instructed by the manufacturer. Analysis of the PI-labeled cells was performed by a flow cytometer (ACCURIBD Biosciences, San Jose, CA, USA) and the cell cycle phases’ distribution was determined as at least 20,000 cells.

### 4.9. Matrigel Invasion Assay

Cell invasion was measured using BD BioCoat Matrigel invasion chambers (BD Biosciences, San Jose, CA, USA, # 354480), as previously described [6]. Briefly, 2.5 × 10^4^ cells were plated in the matrigel-coated 24-well transwell inserts in DMEM-0.5% FBS containing fractions at a fixed concentration. DMEM-10% FBS was used as a chemoattractant. The cells were allowed to invade for 24 h. The invasive cells, attached to the insert membrane, were fixed with methanol and stained with hematoxylin. Then, images were obtained using a × 10 magnification microscope Eclipse 2220 (Nikon) and the cells were counted in all the fields of the membrane. The results are expressed in relation to the DMSO control (considered as 100% of invasion) as the mean percentage of invasion ± SD.

### 4.10. Soft Agar Colony Assay

Cell growth and proliferation of SiHa cell lines under anchorage-independent conditions using the soft agar assay were evaluated as described previously [60]. Briefly, 1 × 10^4^ cells were mixed with an equal volume of 0.6% agar and applied into 6-well plates that had been pre-coated with 0.5 mL of 1.2% agar mixed with the same volume of DMEM-20% FBS. The next day, 5 µg/mL of *A. coriacea* fractions diluted in 0.5 mL of serum-free DMEM were added into the wells, and these treatments were exchanged every two days. Cisplatin (15 µg/mL) was used as a positive control. The cells were allowed to form colonies for 45 days before being fixed with methanol and stained with 0.125% crystal violet. Colonies with more than 50 cells were photographed under the light microscope Eclipse 2200 (Nikon) and the number of colonies was analyzed by open CFU (Plos One—http://opencfu.sourceforge.net/) [61]. The results represent the mean of at least three independent experiments.

### 4.11. Annexin-V-7AAD Assay

This assay was performed using a PE Annexin V Apoptosis Detection Kit (BD Pharmingen, San Diego, CA, USA, #556547) following the manufacturer’s specifications. SiHa cells (2 × 10^5^ cells/mL) were seeded into a six-well plate and treated with 5 and 10 µg/mL of C3 and C5 and cisplatin (15 µg/mL). After the treatment, the cells were harvested and washed with phosphate-buffered saline. Next, 100 µL of each sample were taken and placed into a tube containing 5 µL of FITC Annexin V and 5 µL of 7AAD stain. The suspension was mixed, and 400 µL of 1X Assay buffer were added per tube. All samples were analyzed using a flow cytometer (BD ACCURI™, San Jose, CA, USA).

### 4.12. Analysis of Autophagy Flux

SiHa cells were plated into a six-well plate at a density of 1 × 10^6^ cells/well and allowed to adhere for at least 24 h. Then, the growth medium was replaced with fresh growth medium for control cells, with Hank’s balanced salt solution or Earle’s balanced salts (EBSS, HBSS; Invitrogen, Carlsbad, Califórnia, USA, EUA, # 14155063, # 14025076) for starved cells (two rinses in HBSS or EBSS before being placed in HBSS or EBSS). The cells were then incubated in HBSS and/or C3 and C5 fractions for 24 h using an equivalent concentration to IC_50_ of the evaluated cell line. Then, 20 nM of bafilomycin A1 (Sigma-Aldrich, #B1793) were added to the fraction treatment with EBSS or HBSS as a control condition. Afterward, the cells were scraped into PBS cold and subjected to Western blot analysis as described below.

### 4.13. Acridine Orange Staining

Acidic vacuolar organelles (AVOs) were stained by acridine orange (Sigma-Aldrich, San Luis, MO, USA, EUA, # 235,474 AO) as previously described [62]. Concisely, SiHa cells (2 × 10^5^ cells/mL) were seeded into a six-well plate. After exposure to fractions and bafilomycin (BAF; 10 nM) for 24 h, the cells were trypsinized, harvested, and washed with phosphate-buffered saline. After, the cells were stained with fluorescent dye comprising 10 µL of AO (10 µg/mL). The analysis for AVOs was performed by flow cytometry (BD FACSCanto™ II, San Jose, CA, USA). A minimum of 20,000 cells within the gated region was analyzed.

### 4.14. Detection of Mitochondrial Membrane Potential

MitoStatus Red (BD, Bioscences, San Jose, CA, USA, #564697) was used for the analysis of mitochondrial membrane integrity. SiHa cells (2 × 10^5^) were seeded in 6-well plates. After the cells were treated, fixed doses of the fractions (5, 10 µg/mL) and cisplatin (15 µg/mL) were diluted in culture medium for 24 h at 37 °C. In the end, MitoStatus Red (1 µL/mL) was added, following the manufacturer’s instructions. After incubation, the cells were disaggregated and analyzed by flow cytometry (BD, ACCURI).

### 4.15. Comet Assay

The alkaline comet assay (single-cell gel electrophoresis assay) was performed according to Olive and Banáth [63] with adaptations [64]. Briefly, the HeLa cells were seeded in 24-well plates (2 × 10^5^ cells/well) in complete medium. After 24 h, cells were washed twice with PBS 1X and incubated with the different treatments for 3 h in culture medium without serum. The negative control group was treated with PBS and the positive control group was exposed to methyl methane sulphonate (MMS, 120 µM, Sigma-Aldrich, San Luis, MO, USA, EUA, #129925). The quantification of DNA damage was achieved by visual scoring, with the comets being classified from 0 (no damage) to 4 (maximum damage) [65]. For each treatment, 100 comets were analyzed, and the score of damage was calculated employing the equation: Score = 0(C0) + 1 (C1) + 2(C2) + 3(C3) + 4(C4), where C0–C4 are the numbers of comets in each classification of damage. Three independent experiments were performed and the mean of the scores was calculated for each treatment.

### 4.16. Western Blot

To assess the effect of the drugs on the inhibition of intracellular signaling pathways, the cells were plated at the density of 2 × 10^5^ cells/mL in DMEM-10% FBS into 6-well plates, allowed to grow to 85% of confluence and then serum starved for 2 h, and incubated with IC_50_ values of fractions, diluted in DMEM-0.5% FBS, by 24 h. At the end time, the cells were washed in PBS and lysed with lysis buffer (50 mM Tris (pH 7.6–8), 150 mM NaCl (Sigma-Aldrich, San Luis, MO, USA, EUA, # S9888), 5 mM EDTA (Sigma-Aldrich, San Luis, MO, USA, EUA, # E6758), 1 mM Na_3_VO_4_ (Sigma-Aldrich, San Luis, MO, USA, EUA, # 450243), 10 mM NaF (Sigma-Aldrich, # 201154), 10 mM sodium pyrophosphate (Sigma-Aldrich,# P8010), 1% NP-40 (Sigma-Aldrich, San Luis, MO, USA, EUA, #74385), and 1/7 of protease cocktail inhibitors (Roche, Amadora, Portugal,# 11697498001). Western blot analysis was done using a standard 10% and 15% sodium dodecyl sulfate-polyacrylamide gel electrophoresis, loading 20 µg of protein per lane. All antibodies were provided by cell signaling and used as recommended by the manufacturer (see Table S3 of the Supplementary Materials). Blot detection was done by chemiluminescence (ECL Western Blotting Detection Reagents, #RPN2109; GE Healthcare, Piscataway, NJ, USA) in Image Quant LAS 4000 mini (GE Healthcare).

### 4.17. Statistical Analysis

Single comparisons between the conditions studied were made using Student’s *t*-test, and the differences between the groups were tested using analysis of variance. The statistical analysis was performed using GraphPad Prism version 5. The level of significance in all statistical analyses was set as *p* < 0.05.

## 5. Conclusions

In conclusion, our results showed a comprehensive characterization of antitumor mechanisms associated with *Annona coriacea* Mart. fractions that are cytotoxic in cervical cancer cell lines. Also, we highlight the ability of these fractions to inhibit invasion, clonogenic potential, and autophagy as well as an increase of p21 and subsequent cell cycle arrest in G2/M. All these biological activities observed could be attributed to the alkaloids and acetogenins present in these fractions. Further studies are needed to identify the active substances and to characterize their action using in vitro and in vivo models. Nevertheless, the present findings suggest these compounds are a potential candidate for new drug development for cervical cancer.

## Figures and Tables

**Table 1:** Proposed structures by ESI (-) FT-ICR MS for the main molecules in C3 and C5 fractions from *Annona coriacea.*

*Measured m/z*	*Theoretical m/z*	Error (ppm)	DBE	[M-H]^−^	Proposed Compound	Reference
255.2332	255.23324	−1.12	1	[C_16_H_32_O_2_–H^+^]^−^	palmitic acid	Chen et al., 2016
281.24881	281.24886	0.92	2	[C_18_H_34_O_2_–H^+^]^−^	oleic acid	Chen et al., 2016
595.45815	595.45822	−0.49	4	[C_35_H_64_O_7_–H^+^]^−^	asitrocinone	Adewole e Ojewole et al., 2008
595.45838	595.45845	−0.87	4	[C_35_H_64_O_7_–H^+^]^−^	annonacin	Alkofahi et al., 1988
609.43885	609.43893	−2.85	5	[C_35_H_62_O_8_–H^+^]^−^	trilobalicin	He et al., 1997
611.45312	611.45314	−0.49	4	[C_35_H_64_O_8_–H^+^]^−^	annomuricin E	Kim et al., 1998
621.4742	621.4743	−1.17	5	[C_37_H_66_O_7_–H^+^]^−^	asimicin	Ye et al., 1996
621.47413	621.47418	−0.96	5	[C_37_H_66_O_7_–H^+^]^−^	bullatacin	Morre et al., 1995
627.4483	627.44832	−0.9	4	[C_35_H_64_O_9_–H^+^]^−^	annohexocin	Moghadamtousi et al., 2015
627.44823	627.44828	−0.83	4	[C_35_H_64_O_9_–H^+^]^−^	murihexocin	Kim et al., 1998
635.4540	635.4542	−2.14	6	[C_37_H_64_O_8_–H^+^]^−^	goniotriocin	Alali et al., 1999
637.46921	637.46927	−1.22	5	[C_37_H_66_O_8_–H^+^]^−^	bullatalicinone	Hui et al., 1991
637.46905	637.46914	−1.02	5	[C_37_H_66_O_8_–H^+^]^−^	annoglaucin	Bermejo et al., 2005
641.42889	641.42895	−1.34	4	[C_35_H_64_O_10_–H^+^]^−^	coriaheptocin B/A	Formagio et al., 2015
651.44943	651.44949	−2.65	6	[C_35_H_64_O_10_–H^+^]^−^	ginsenoside Rh5	Vamanu, 2014
653.46442	653.46444	−1.58	5	[C_37_H_66_O_9_–H^+^]^−^	salzmanolin	Queiroz et al., 2003
669.46005	669.4601	−1.22	6	[C_37_H_68_O_10_–H^+^]^−^	annoheptocin A	Meneses Da Silva et al., 1998
671.47569	671.47575	−1	6	[C_37_H_68_O_10_–H^+^]^−^	annoheptocin B	Meneses Da Silva et al., 1998
763.47932	763.47939	−0.83	12	[C_39_H_70_O_5_–H]^−^	squamocin glycosilated	Jamkhande e Wattamwar, 2015

DBE: Double bond equivalent; m/z: mass-to-charge ratio.

**Table 2:** IC_50_ values for *A. coriacea* compounds and cisplatin in cervical cancer cell lines.

IC_50_ Value (Mean ± SD) µg/mL								
Cell Line	C1	C2	C3	C4	C5	C6	C7	Cisplatin
CaSki	17.8 ± 2.8	ND	6.5 ± 1.8	ND	3.6 ± 0.9	11.7 ± 2.2	21.4 ± 3.3	1.05 ± 1.2
HeLa	12.2 ± 1.5	ND	6.6 ± 1.2	ND	4.1 ± 0.4	12.9 ± 1.9	12.3 ± 0.83	13.6 ± 0.44
SiHa	16.1 ± 2.7	ND	8.7 ± 1.3	ND	5.1 ± 0.6	12.6 ± 1.6	12.7 ± 1.3	15.5 ± 0.93

ND: Not determined; C1: Ethanolic extract; C2: Hexane fraction; C3: Ethyl acetate fraction; C4: Hidroalcoholic fraction; C5: Fraction enriched in acetogenin; C6: Neutral dichloromethane fraction obtained from acid-base extraction; C7: Dichloromethane fraction enriched in alkaloids.

**Table 3:** IC_50_ values and selectivity index for the C3 and C5 fractions of cisplatin to tumor cells as compared with HaCaT.

IC50 Value (Mean ± SD) µg/mL and SI ª						
Cell Line	C3	C5	Cisplatin	SIC3	SIC5	SI Cisplatin
CaSki	6.5 ± 1.8	3.6 ± 0.9	1.05 ± 1.2	1.57	3.72	4.57
HeLa	6.6 ± 1.2	4.1 ± 0.4	13.6 ± 0.44	1.55	3.27	0.35
SiHa	8.7 ± 1.3	5.1 ± 0.6	15.5 ±0.93	1.17	2.63	0.31
HaCat	10.2 ± 2.4	13.4 ± 1.0	4.8 ± 1.3	R	R	R

^a^ Selectivity index is the ratio of the IC_50_ values of the treatments on HaCaT cells to those in the cancer cell lines. SI: Selectivity index; C3: Ethyl acetate fraction; C5: Fraction enriched in acetogenin; R: Reference cell line.

## Supplementary Materials

The following are available online, Supplementary Table S1. Antibodies used in Western Blot analysis; Supplementary Table S2. -Summary all *Annona coriacea* Mart fractions used in the present study; Supplementary Table S3. -IC_50_ values for the *A. coriacea* fractions in kinectics assay on cervical cell lines.
