# Supplementary material for: Annona coriacea Mart. Fractions Promote Cell Cycle Arrest and Inhibit Autophagic Flux in Human Cervical Cancer Cell Lines
Source: Molecules. 2019 Nov 1;24(21):3963. doi: 10.3390/molecules24213963 (PMC6864525; doi:10.3390/molecules24213963)
Supplement: Supplementary file 1 [file molecules-24-03963-s001.pdf]

Supplementary table 1 - Summary of *Annona coriacea* Mart fractions

Name	Fractions
C1	Ethanolic extract
C2	Hexane fraction
C3	Ethyl-acetate fraction
C4	Hydroalcoholic fraction
C5	Fraction enriched in acetogenin
C6	Neutral dicholoromethane fraction obtained acid-base extraction
C7	Dicholoromethane fraction enriched in alkaloids

Supplementary table 2- IC_{50} values for the *A. coriacea* fractions in Kinectics assay on cervical cell lines

	**IC_{50} values of C3 and C5 (Mean ±SD) µg/mL**							
Time (Hrs)	0	3	6	12	16	24	36	72
Cell line	C3/C5	C3/C5	C3/C5	C3/C5	C3/C5	C3/C5	C3/C5	C3/C5
CaSki	ND	19.05 ± 2.9/ 24.4 ± 0.1	15.1 ± 0.2/18.7 ± 0.7	15.5 ± 1.8/15.5 ± 5.9	18.5 ± 3.1/14.0 ± 5.1	13.3 ± 2.2/8.9 ± 0.9	9.3 ± 0.5/7.06 ± 1.6	6.5 ± 1.8/3.6 ± 0.9
HeLa	ND	ND	25.0 ± 2.8/23.8 ± 3.5	19.0 ± 7.3/18.2 ± 6.2	21.8 ± 0.0/17.3 ± 6.9	14.3 ± 1.01/12.4 ± 3.3	11.6 ± 0.5/8.3 ± 0.8	6.6 ± 1.2/4.1 ± 0.4
SiHa	ND	23.7 ± 0.1/19.05 ± 1.0	22.9 ± 0.2/18.9 ± 4.7	19.2 ± 3.6/ 18.7 ± 1.7	21.7 ± 0.6/20.5 ± 3.6	14.9 ± 1.3/12.7 ± 4.3	11.6 ± 1.8/10.2 ± 2.3	8.7 ± 1.3/5.1 ± 0.6

ND- Not determined; C1: Ethanolic extract; C2: Hexane fraction; C3: Ethyl acetate fraction; C4: Hidroalcoholic fraction; C5: Fraction enriched in acetogenin; C6: Neutral dichloromethane fraction obtained from acid-base extraction; C7: Dichloromethane fraction enriched in alkaloids

Supplementary table 3- Antibodies used in Western Blot analysis

Antibody	Diluition	Condition	Manufacturer / Code
Anti - β actin	1/1000	4ºC- Overnight	Cell signaling/ #4967
Anti- PARP (total)	1/1000	4ºC- Overnight	Cell signaling/ #9542
Anti- Caspase 3 (total)	1/1000	4ºC- Overnight	Cell signaling/ #9662
Anti-Histon H2A.X (fosforilado)	1/1000	4ºC- Overnight	Cell signaling/ #9718
Anti-Histon H2A.X (total)	1/1000	4ºC- Overnight	Cell signaling/ #2595
Anti- BAX	1/1000	4ºC- Overnight	Cell signaling/ #2772
Anti- Caspase 7 (total)	1/1000	4ºC- Overnight	Cell signaling/ #9492
Anti- Caspase 7 (clivada)	1/1000	4ºC- Overnight	Cell signaling/ #9491
Anti- AKT (fosforilado)	1/1000	4ºC- Overnight	Cell signaling/ #9271
Anti- AKT (total)	1/1000	4ºC- Overnight	Cell signaling/ #9272
Anti- p44/42 MAPK (fosforilado)	1/1000	4ºC- Overnight	Cell signaling/ #9101
Anti- p44/42 MAPK (total)	1/1000	4ºC- Overnight	Cell signaling/ #9102
Anti- p27 Kip1	1/1000	4ºC- Overnight	Cell signaling/ #2552
Anti- p21 Waf1/Cip1	1/1000	4ºC- Overnight	Cell signaling/ #2947
Anti- LC3A/B (D3U4C)	1/1000	4ºC- Overnight	Cell signaling/ #12741
Anti-mouse IgG HPR	1/5000	TA- 1 hour	Cell signaling/ #7076
Anti-rabbit IgG HPR	1/5000	TA- 1 hour	Cell signaling/ #7074
